# Brain functional connectivity in hyperthyroid patients: systematic review

**DOI:** 10.3389/fnins.2024.1383355

**Published:** 2024-04-24

**Authors:** Ephrem Tesfaye, Mihret Getnet, Desalegn Anmut Bitew, Dagnew Getnet Adugna, Lemlemu Maru

**Affiliations:** ^1^Department of Biomedical Sciences, Madda Walabu University Goba Referral Hospital, Bale-Robe, Ethiopia; ^2^Department of Human Physiology, School of Medicine, College of Medicine and Health Science, University of Gondar, Gondar, Ethiopia; ^3^Department of Epidemiology and Biostatistics, Institute of Public Health, College of Medicine and Health Science, University of Gondar, Gondar, Ethiopia; ^4^Department of Reproductive Health, Institute of Public Health, College of Medicine and Health Science, University of Gondar, Gondar, Ethiopia; ^5^Department of Anatomy, School of Medicine, College of Medicine and Health Science, University of Gondar, Gondar, Ethiopia

**Keywords:** brain, fMRI, functional connectivity, hyperthyroid, resting-state fMRI

## Abstract

**Introduction:**

Functional connectivity (FC) is the correlation between brain regions’ activities, studied through neuroimaging techniques like fMRI. It helps researchers understand brain function, organization, and dysfunction. Hyperthyroidism, characterized by high serum levels of free thyroxin and suppressed thyroid stimulating hormone, can lead to mood disturbance, cognitive impairment, and psychiatric symptoms. Excessive thyroid hormone exposure can enhance neuronal death and decrease brain volume, affecting memory, attention, emotion, vision, and motor planning.

**Methods:**

We conducted thorough searches across Google Scholar, PubMed, Hinari, and Science Direct to locate pertinent articles containing original data investigating FC measures in individuals diagnosed with hyperthyroidism.

**Results:**

The systematic review identified 762 articles, excluding duplicates and non-matching titles and abstracts. Four full-text articles were included in this review. In conclusion, a strong bilateral hippocampal connection in hyperthyroid individuals suggests a possible neurobiological influence on brain networks that may affect cognitive and emotional processing.

**Systematic Review Registration:**

PROSPERO, CRD42024516216.

## Introduction

Functional connectivity (FC) refers to the statistical correlation between the activities of different brain regions, typically observed through neuroimaging techniques such as functional magnetic resonance imaging (fMRI) (Müller, 2013; Cao et al., 2022; Ursino et al., 2022). Studies of it aim to understand how different brain regions communicate and coordinate their activities during various cognitive processes or in different states (Cao et al., 2022). Analyses of it have become increasingly important in neuroscience, offering valuable information about brain function, organization, and dysfunction. Researchers use these analyses to explore normal brain function, investigate neurological and psychiatric disorders, and assess the effects of interventions or treatments on brain connectivity patterns (Wojtalik et al., 2018).

Hyperthyroidism is defined as a high serum level of free thyroxin (FT4) and/or triiodothyronin (T3) and a suppressed thyroid stimulating hormone (TSH) level (Samuels, 2014; Ross et al., 2016). Thyroid hormone (TH) is essential for normal brain development and may also promote recovery and neuronal regeneration after brain injury (Liu and Brent, 2018; Talhada et al., 2019). Thyroid hormones are essential for appropriate growth, reproduction, and regulation of energy metabolism, neuronal development, and cognitive and behavioral development (Stasiolek, 2015; Taylor et al., 2018; Mathew et al., 2020). The mechanisms include the regulation of neuronal plasticity processes, stimulation of angiogenesis and neurogenesis, as we as modulating the dynamics of cytoskeletal elements, and intracellular transport processes (Talhada et al., 2019).

It is clear that without optimal thyroid function, mood disturbance, cognitive impairment, and other psychiatric symptoms can emerge (Lekurwale et al., 2023). In animal studies, changes in the release pattern of acetylcholine and monoamines have been found in the hippocampus and frontal cortex of experimentally induced hyperthyroid rats, along with associated functional changes (Eslami-Amirabadi and Sajjadi, 2021). Particularly in severe cases, thyroid dysfunction can result in a variety of emotional and cognitive disorders, such as executive function deficiencies, depression, anxiety, and irritability (Samuels, 2014; Stasiolek, 2015).

Related to the morphological changes of hyperthyroid individuals in the brain, exposure to excess thyroid hormones has been shown to enhance neuronal death and decrease brain volume (Folkestad et al., 2020), which leads to more severe atrophy of the amygdala (Wu et al., 2016; Eslami-Amirabadi and Sajjadi, 2021) and hippocampus (Wu et al., 2016; Eslami-Amirabadi and Sajjadi, 2021; Quinlan et al., 2022). Hyperthyroid patients exhibited reduced grey matter volume in regions associated with memory, attention, emotion, vision, and motor planning (Zhang et al., 2014).

The exploration of functional connectivity between brain regions is deemed essential to elucidate the neuropsychiatric symptoms associated with hyperthyroidism and the impact of elevated thyroid hormone levels on the adult brain (Cao et al., 2022; Lekurwale et al., 2023). Thyroid hormones play a crucial role in functional connectivity under physiological conditions (Schroeder and Privalsky, 2014). In the brain, T4 is converted to active T3 by type 2 deiodinase produced by glial cells, highlighting the importance of these hormones in brain development and function (Fingeret, 2024). Studies revealed functional connectivity changes in hyperthyroid patients, an increase in functional connectivity in the rostral temporal lobes, which is integrated with the cognitive control network (Göbel et al., 2020), lower amplitude of low-frequency fluctuations (ALFF) was found in the patients in the right posterior cingulate cortex, and increased functional connectivity in the bilateral anterior and posterior insula, and importantly, in the left anterior lobe of the cerebellum (Göbel et al., 2020). Research has shown that thyroid hormone functions may play a crucial role in modulating functional connectivity in early-course schizophrenia, impacting cognition and functional outcomes (George et al., 2023), resting-state brain network functional connectivity, and shedding light on the intricate relationship between thyroid function and brain network dynamics (Li et al., 2022).

Despite the significance of certain brain regions in emotional and cognitive regulation, there is a notable gap in research pertaining to the interactions between and within these regions in hyperthyroid patients. This review highlights hyperthyroidism’s potential impact on connectivity between brain regions and improves our understanding of the functional connectivity of targeted regions.

## Method

### Registration and protocol

This study protocol is registered with the International Prospective Register of Systematic Reviews website (PROSPERO; registration number CRD42024516216).

### Eligibility criteria

**Hyperthyroid patients:** all patients who have elevated serum FT3 or FT4 levels, and decreased TSH levels (Ross et al., 2016; Toyib et al., 2019).

**Pre/post studies:** one experimental session was performed before and one after the end of administration of medications or procedures to assess the impact of medications like anti-thyroid drugs, radioiodine therapy, beta blockers, and thyroidectomy on patients with hyperthyroidism (Doubleday and Sippel, 2020).

We applied the PICO method as a selection criteria for articles:

**Population**: hyperthyroid patients.

**Interventions:** thyroid hormone thyroxin replacement therapies, for example, levothyroxine.

**Study type**: randomized controlled trials, case–control studies, and quasi-experimental studies.

**Cases**: hyperthyroid patients.

**Control**: healthy controls.

**Outcomes**: primary outcome– brain functional connectivity.

**Outcome assessment time**: There was no limit to the outcome assessment time.

**Publication year and language**: English-language literature, with publication year not limited. **List of countries**: all countries in the world.

### Search strategy and selection criteria

Four databases– PubMed, Hinari, Science Direct, and Google Scholar– were used to identify studies about brain functional connectivity from the inception date to November 21, 2023. Using title, abstract, and keywords, we searched out the primary studies using the keywords selected: brain, connectivity, network, hyperthyroidism, and their synonyms using AND, OR, and NOT filters as described in Supplementary file 2. This systematic review was prepared according to the instructions of the PRISMA guideline.

### Data extraction

We developed a form to extract the suitable data, including the following details: (1) characteristics of the papers (authors, publication year, and country); (2) characteristics of the participants (sample size, age range, and drug use); (3) study design and measurement method; (4) method of analysis; and (5) results. Two authors (ET and LM) independently extracted the data, and disagreements were resolved by discussing with the third author (MG).

## Results

### Identification of eligible studies

**Figure 1:**
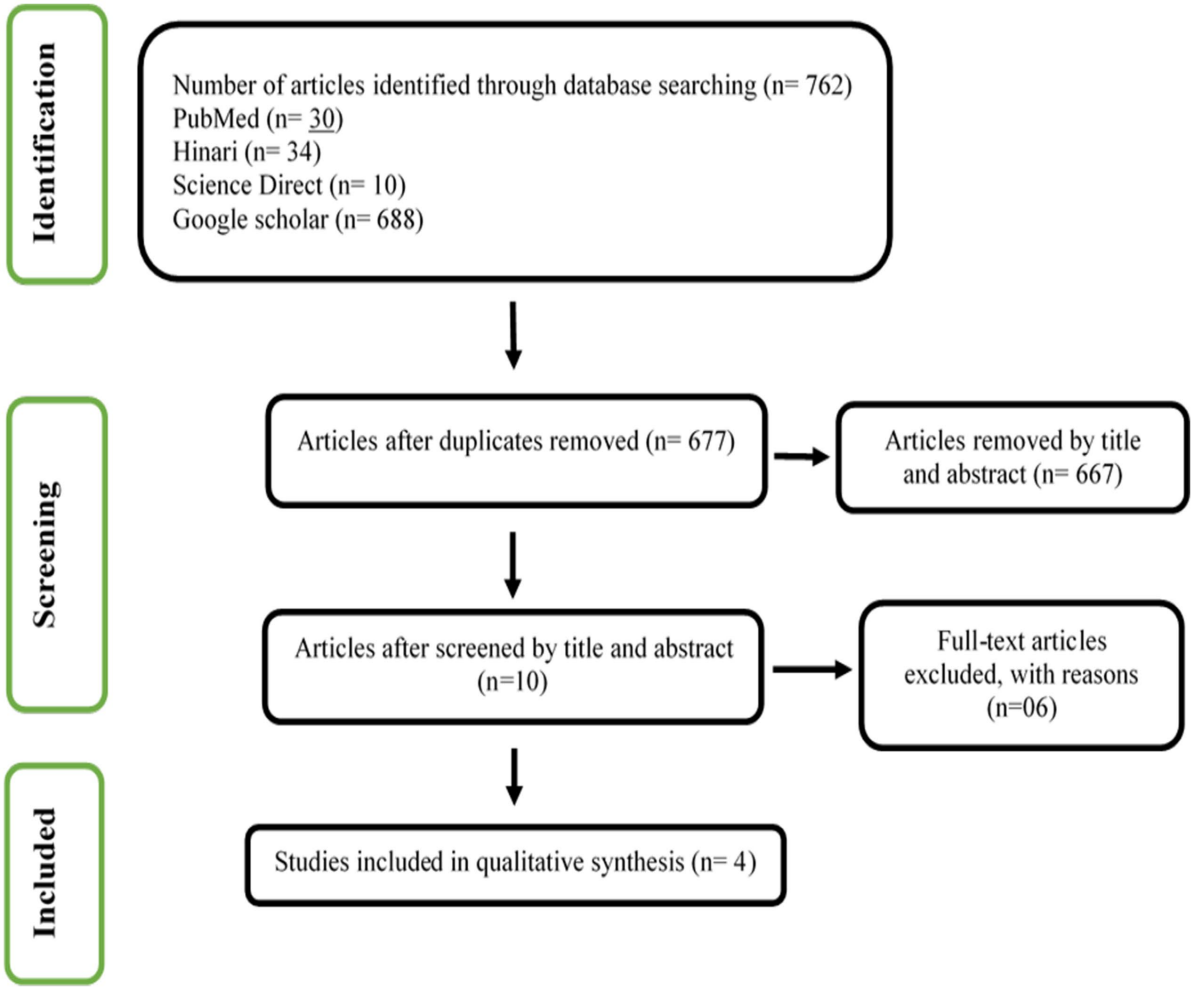
PRISMA flow chart for selection of eligible articles.

Figure 1 shows the result of our screening process. We identified 762 articles with our searching strategy. Duplicate articles (*n* = 85) were excluded. The articles that according to title and abstract did not match the selection criteria (*n* = 667) were also excluded. Finally, four articles out of 10 available full-text articles were included in this systematic review. The details of the excluded six articles are presented in Supplementary file S1.

### Characteristics of included studies

The included studies were either case–control or quasi-experimental studies. The etiology of the disease in the three studies was Graves’ disease, and one drug-induced pre-and post-study. They were all small studies, with the largest sample size of 47. General characteristics of the studies, like the first author’s name, year of publication, country, sample size (case/control or pre-post), age range of participants, and drug use for the study, are shown in Table 1, and the imaging method, study design, analysis method, and results are presented in Table 2.

**Table 1:** General characteristics of the studies included in this systematic review.

Author/year	Country	Patient’s characteristics			
		Sample size (n)	Age in years (Mean ± SD)	Duration of disease/ Rx	Etiology of disease
Zhang et al. (2014)	China	-Cases: 46 -Controls: 46	-Case: 29.72 ± 7.93 -Controls: 29.26 ± 6.50	8.74 ± 5.64 months	Graves’ disease
Göttlich et al. (2015)	German	*n* = 29	30 (21—49)	Levothyroxine 250 μg per day for 8 weeks	Drug induced
Zhang et al. (2018)	China	-Cases: 13 hyperthyroid pts. -Controls: 13 healthy	-Case: 32.7 ± 10.2 -Controls: 33.2 ± 11.3	–	Graves’ disease
Li et al. (2017)	China	-Case: 33 hyperthyroid -Controls: 33 HC	-case: 37.36 ± 12.43 -control: 39.03 ± 13.28	9.94 ± 17.31 months	Graves’ disease

**Table 2:** List of studies with their methods and results.

Author/year	Imaging method	Study design	Analysis methods	Result
Zhang et al. (2014)	rs-fMRI	Case–control	Seed voxel correlation approach	Within-group analysis: -The bilateral hippocampus showed strong connectivity to other regions in the bilateral limbic system (hippocampus, parahippocampal gyrus, amygdala, and insula), bilateral temporal lobe (inferior/middle/superior temporal gyrus and temporal pole), thalamus, bilateral basal ganglia (globus pallidus, caudate, and putamen), bilateral frontal lobe (medial/inferior frontal gyrus, orbital frontal cortex, ACC), brainstem, and bilateral cerebellum. Between-group analysis: -The bilateral ACC and PCC showed significantly weaker connectivity to the left hippocampus in the hyperthyroid group. -The hyperthyroid group showed a reduced connection between the bilateral ACC, bilateral PCC, and right medial orbitofrontal cortex (mOFC) with the right hippocampus. Correlation between functional connectivity and clinical variables: -When the seed was located in the left hippocampus, there was a significant negative correlation between disease duration and the strength of FC to both the bilateral ACC and bilateral PCC. -Similarly, when the seed was placed in the right hippocampus, significant negative correlations were found between disease duration and FC strength to both the bilateral ACC and PCC.
Göttlich et al. (2015)	rs-fMRI	Quasi-experimental	-Voxel degree centrality maps -Seed-based functional connectivity	-Increase in degree centrality in the right inferior temporal gyrus, left middle temporal gyrus, right middle temporal gyrus, and left middle temporal pole. -Significantly increase functional connectivity in the bilateral temporal poles and left middle temporal gyrus. -The left temporal pole was significantly stronger and connected to the dorsal anterior cingulate cortex (dACC), inferior temporal gyrus (ITG), inferior frontal gyrus (IFG), middle frontal gyrus (MFG), and supramarginal gyrus (SMG). -The right temporal pole showed significantly stronger connectivity to the MFG, IFG, and SMG.
Zhang et al. (2018)	rs-fMRI	Case–control	-ALFF analysis -region of interest (ROI) -based functional connectivity analysis	-Decreased ALFF values in the patient group included the posterior cingulate gyrus and bilateral inferior parietal gyrus. -Increased ALFF values in the right thalamus and bilateral cuneus -Significant negative correlation between ALFF values of the left inferior parietal gyrus and the left posterior cingulate gyrus -ROI-based FC analysis revealed increased FCs between the left inferior parietal gyrus and left rostral ACC and bilateral frontal lobe; left posterior cingulate gyrus and bilateral left temporal lobe.
Li et al. (2017)	rs-fMRI	Case–control	-Degree centrality -Seed-based connectivity analyses	-Hyperthyroid patients had decreased degree centrality values in the left posterior lobe of the cerebellum and bilateral medial frontal gyrus. -Decreased functional connectivity between seed-1 located in the left posterior lobe of the cerebellum (PLC) and right middle temporal gyrus (MTG) in the attention network. -Lowered functional connectivity from both the left PLC and right cerebellum to the medial frontal gyrus (MeFG).

## Discussion

Reviewing the available evidence, we find significant changes in brain functional connectivity among hyperthyroid patients. These alterations imply that hyperthyroidism may impact brain networks neurobiologically. Studying connectivity patterns in healthy individuals and those with hyperthyroidism can help us understand disruptions in thyroid dysfunction networks, clarify cognitive and emotional symptoms in thyroid disorders, and guide future therapeutic interventions targeting neural circuits. In hyperthyroid patients, alterations in functional connectivity have been observed, particularly in regions associated with emotion regulation, memory, and cognitive processing (Chen et al., 2021). Changes in FC observed in hyperthyroid patients can be attributed to several mechanisms and could explain the manifestations of different disorders.

Recent advancements in neuroimaging techniques have shed light on the intricate neural alterations accompanying this disorder (Minnerop et al., 2018; Yen et al., 2023). Among these, changes in FC within the brain have emerged as a critical area of investigation. The observed connectivity between hyperthyroid patients and healthy controls suggests shared neural circuitry, potentially crucial for detecting the hippocampal memory system’s operation in humans (Liu et al., 2017; Ma et al., 2022).

One of the central findings of the reviewed papers is the disruption in connectivity patterns involving the hippocampus and cingulate cortex. Zhang et al’s. (2014) study found that hyperthyroid individuals show weakened connectivity between the bilateral ACC and PCC and the hippocampi. This suggests that hyperthyroidism affects the limbic system, which is crucial for memory consolidation and emotional regulation. The alterations may indicate cognitive or mental disorders associated with the hippocampus and other brain areas (Li et al., 2017; Yao et al., 2022). Hyper-connectivity patterns may affect the functional connectivity of the default mode network, potentially impacting episodic memory and self-representation (Zanão et al., 2017; Staffaroni et al., 2018; Ursino et al., 2022). The direct effects of thyroid hormones on these brain regions contribute to their functional integrity and connectivity (Biswas and Dey, 2014). Thyroid hormones have receptors in the cingulate cortices and hippocampi. T3 and T4 influence neurotransmitter systems such as glutamate (Ritchie and Yeap, 2015; Zhu et al., 2022), and gamma-aminobutyric acid (GABA) (Yi et al., 2014; Prisciandaro et al., 2021), which are crucial for synaptic transmission and neuronal plasticity in the cingulate cortices (Prisciandaro et al., 2021). Alterations in thyroid hormone levels can disrupt the balance of excitatory and inhibitory neurotransmission, leading to changes in neural connectivity and function within the ACC and PCC and impairing hippocampal function, leading to deficits in memory consolidation, emotional processing, and spatial navigation (Bavarsad et al., 2019).

Moreover, the correlation between FC strength and clinical variables provides valuable insights into the progression of the disease (Zhang et al., 2014). A significant negative correlation was found between disease duration and FC strength between the hippocampi and cingulate cortices (Zhang et al., 2014; Milton et al., 2022). This suggests that as the disease progresses, there is a decline in the integrity of neural circuits linking these regions (Zhi et al., 2018; Johansson et al., 2023), due to adaptive changes or neuronal damage in hyperthyroid patients. In addition, chronic hyperthyroidism could lead to structural (Zhang et al., 2014; Zhe et al., 2021; Duda et al., 2023; Xiong et al., 2023), and functional changes in the hippocampi and cingulate cortices, affecting their connectivity patterns. This is clinically important in identifying neuroimaging markers that can be used to track the progression of hyperthyroidism and assess the effectiveness of treatment interventions (Clerc, 2020).

Beyond hippocampal-cingulate alterations, hyperthyroidism is also associated with changes in FC involving regions crucial for cognitive processing and emotional regulation (Göttlich et al., 2015). Increased degree centrality was observed in temporal regions, including the right inferior temporal gyrus, left middle temporal gyrus, right middle temporal gyrus, and left middle temporal pole. Additionally, there was a significant increase in FC within the bilateral temporal poles and left middle temporal gyrus (Göttlich et al., 2015; Zhang et al., 2018), indicating heightened connectivity within temporal regions. Notably, the left temporal pole exhibited stronger connections with various regions, including the dACC, ITG, and frontal gyrus, underscoring the widespread impact of hyperthyroidism on functional brain networks. Degree centrality refers to the number of connections a node (brain region) has with other nodes in the network (Yoo et al., 2017; Jia et al., 2019). The heightened degree centrality indicates increased functional connectivity and communication within these temporal regions. This indicates increased synchronization and information exchange within these regions (Csató, 2017; Lorenzini et al., 2022). The study suggests that hyperthyroid patients’ cognitive deficits may be linked to disrupted functional coordination within the default mode network (DMN), emphasizing the significance of interhemispheric connectivity (Zhi et al., 2018; Berron et al., 2020; Wang et al., 2023).

Conversely, decreased ALFF was noted in regions such as the posterior cingulate gyrus and bilateral inferior parietal gyrus (Zhang et al., 2018), suggesting reduced neural activity. In association with this, Milton et al.’s ROI-based functional connectivity analysis reveals changes in connectivity patterns in the inferior parietal gyrus and posterior cingulate gyrus, indicating complex regional dynamics (Uddin et al., 2009). Additionally, disruptions in FC were observed in cerebellar-frontal circuits, with decreased connectivity between the left PLC and MTG within the attention network (Li et al., 2017). Besides its motor coordination ability, the cerebellum increasingly recognized for its role in cognitive functions, including attention (Li et al., 2017; Liu et al., 2017; Yao et al., 2022). Dysfunction within the cerebellum and frontal regions impairs the coordination and modulation of attention networks (Arif et al., 2020). Damage to the tract and disruptions in neuronal synchronization between the cerebellum and frontal cortex may contribute to decreased functional connectivity (Wang et al., 2023). Cognitive ability is affected by reduced connectivity between cortical regions, particularly the prefrontal cortex, and sub-cortical regions in schizophrenia (Sheffield and Barch, 2016), bipolar disease (Ursino et al., 2022), depression (Liu et al., 2020), traumatic brain injury (Morelli et al., 2021; Nakuci et al., 2021), stroke (Wang et al., 2023), and functional seizure (Foroughi et al., 2020).

Taken together, these findings highlight the complex nature of the brain changes linked to hyperthyroidism. The dysregulation of thyroid hormones affects multiple pathways and mechanisms within the brain, leading to diverse neurological manifestations. This complexity underscores the need for a comprehensive understanding and management of the neurological aspects of hyperthyroidism. The findings open the door for additional research into the functional implications of these connectivity changes and how they might impact the mental and emotional health of hyperthyroid patients, in addition to expanding our understanding of the brain mechanisms underlying thyroid dysfunction (Ritchie and Yeap, 2015; Eslami-Amirabadi and Sajjadi, 2021). Combining these many viewpoints allows for a more thorough understanding of the complex relationship between thyroid function and brain connections.

### Limitations

This systematic review had some limitations.

- The exploration of functional connectivity in neuroscience has encountered limitations, with a paucity of comprehensive studies on the intricate networks that govern brain function. To advance our understanding of the dynamic relationships between distinct brain regions, there is a pressing need for more extensive studies on brain functional connectivity in patients with hyperthyroidism.
- A significant limitation frequently encountered in research is the small sample size. Small sample sizes can magnify individual differences and chance variations, making it challenging to draw robust conclusions or to establish the true effect of an intervention or phenomenon. The studies included in this review had a small sample size, with a minimum of 13 and a maximum of 46.
- All studies used fMRI as the imaging technique. It has limitations compared to other connectivity techniques, including lower temporal resolution (vs. EEG/MEG), sensitivity to motion artifacts, and reliance on blood flow measurement. Techniques like EEG and MEG offer better temporal resolution.

## Conclusion and recommendation

In conclusion, research on brain functional connectivity among patients with hyperthyroidism suggests a potential neurobiological impact of hyperthyroidism on intricate brain networks. This study found strong bilateral hippocampal connectivity across various brain regions, suggesting a fundamental neural network. Alterations in connectivity patterns suggest a potential hub role in hyperthyroid states, affecting cognitive and emotional processing. These findings highlight the complex nature of brain changes linked to hyperthyroidism and suggest the need for further investigations into the functional effects of these connectivity alterations on mental and emotional well-being.

We suggest exploring how changes in connectivity affect thinking and emotions in hyperthyroidism patients to help develop better mental health treatments. Furthermore, given the recognized challenge of small sample sizes in research, it is advisable for future studies to strive for larger and more representative samples to enhance the reliability and generalizability of the findings. Additionally, researchers should consider diversifying imaging techniques beyond fMRI to overcome its limitations such as lower temporal resolution and susceptibility to motion artifacts.

## Data availability statement

The original contributions presented in the study are included in the article/Supplementary material, further inquiries can be directed to the corresponding author.

## Author contributions

ET: Conceptualization, Data curation, Formal analysis, Writing – original draft, Writing – review & editing. MG: Conceptualization, Data curation, Methodology, Supervision, Writing – original draft, Writing – review & editing. DeA: Conceptualization, Data curation, Methodology, Writing – original draft, Writing – review & editing. DaA: Conceptualization, Data curation, Formal analysis, Writing – original draft, Writing – review & editing. LM: Conceptualization, Data curation, Formal analysis, Writing – original draft, Writing – review & editing.
