# Supplementary material for: Brain functional connectivity in hyperthyroid patients: systematic review
Source: Front Neurosci. 2024 Apr 24;18:1383355. doi: 10.3389/fnins.2024.1383355 (PMC11080614; doi:10.3389/fnins.2024.1383355)
Supplement: Supplementary file 1 [file Table_1.docx]

**Search terms**

**Hinari**

- (brain connectivity) AND (hyperthyroidism)
- (brain network) AND (hyperthyroidism)

**PubMed**

- (Bain) AND (connect) AND (hyperthyroid) NOT (systematic review) NOT (review)

(((("brain"[MeSH Terms] OR "brain"[All Fields] OR "brains"[All Fields] OR "brain s"[All Fields]) AND ("connect"[All Fields] OR "connectable"[All Fields] OR "connected"[All Fields] OR "connecting"[All Fields] OR "connection"[All Fields] OR "connectional"[All Fields] OR "connections"[All Fields] OR "connective"[All Fields] OR "connectives"[All Fields] OR "connectivities"[All Fields] OR "connectivity"[All Fields] OR "connects"[All Fields] OR "connexion"[All Fields] OR "connexions"[All Fields]) AND ("hyperthyroidal"[All Fields] OR "hyperthyroidic"[All Fields] OR "hyperthyroidism"[MeSH Terms] OR "hyperthyroidism"[All Fields] OR "hyperthyroid"[All Fields] OR "hyperthyroids"[All Fields] OR "hyperthyroidisms"[All Fields])) NOT ("review"[Publication Type] OR "review literature as topic"[MeSH Terms] OR "review"[All Fields])) NOT ("systematic review"[Publication Type] OR "systematic reviews as topic"[MeSH Terms] OR "systematic review"[All Fields]))

- (brain*) AND (connect*) AND (hyperthyroid*) NOT (systematic review) NOT (review)

(("brain*"[All Fields] AND "connect*"[All Fields] AND "hyperthyroid*"[All Fields]) NOT ("systematic review"[Publication Type] OR "systematic reviews as topic"[MeSH Terms] OR "systematic review"[All Fields])) NOT ("review"[Publication Type] OR "review literature as topic"[MeSH Terms] OR "review"[All Fields])

**Science Direct**

- Brain AND network

**Google Scholar**

- Brain, functional, connectivity, "hyperthyroid"
- "brain" AND "functional" AND "connectivity" AND "hyperthyroid"
